# Autonomic nervous system changes detected with peripheral sensors in the setting of epileptic seizures

**DOI:** 10.1038/s41598-020-68434-z

**Published:** 2020-07-14

**Authors:** Solveig Vieluf, Claus Reinsberger, Rima El Atrache, Michele Jackson, Sarah Schubach, Claire Ufongene, Tobias Loddenkemper, Christian Meisel

**Affiliations:** 1 Division of Epilepsy and Clinical Neurophysiology, Boston Children’s Hospital, Harvard Medical School, 300 Longwood Ave, Boston, MA 02115 USA; 2 Institute of Sports Medicine, Paderborn University, Warburger Str. 100, 33098 Paderborn, Germany; 3 Edward E. Bromfield Epilepsy Center, Brigham and Women’s Hospital, Harvard Medical School, 75 Francis Street, Boston, MA 02115 USA; 4 Department of Neurology, University Clinic Carl Gustav Carus, Fetscherstraße 74, Dresden, 01307 Germany

**Keywords:** Neurology, Epilepsy

## Abstract

A better understanding of the early detection of seizures is highly desirable as identification of an impending seizure may afford improved treatments, such as antiepileptic drug chronotherapy, or timely warning to patients. While epileptic seizures are known to often manifest also with autonomic nervous system (ANS) changes, it is not clear whether ANS markers, if recorded from a wearable device, are also informative about an impending seizure with statistically significant sensitivity and specificity. Using statistical testing with seizure surrogate data and a unique dataset of continuously recorded multi-day wristband data including electrodermal activity (EDA), temperature (TEMP) and heart rate (HR) from 66 people with epilepsy (9.9 ± 5.8 years; 27 females; 161 seizures) we investigated differences between inter- and preictal periods in terms of mean, variance, and entropy of these signals. We found that signal mean and variance do not differentiate between inter- and preictal periods in a statistically meaningful way. EDA signal entropy was found to be increased prior to seizures in a small subset of patients. Findings may provide novel insights into the pathophysiology of epileptic seizures with respect to ANS function, and, while further validation and investigation of potential causes of the observed changes are needed, indicate that epilepsy-related state changes may be detectable using peripheral wearable devices. Detection of such changes with wearable devices may be more feasible for everyday monitoring than utilizing an electroencephalogram.

## Introduction

The current inability to assess when a seizure is most likely to occur constitutes a major burden for people with epilepsy (PWE)^1^. From a clinical perspective, this inability precludes the development of better treatments, such as antiepileptic drug chronotherapy adapted to personalized risk profiles, or timely, closed-loop intervention strategies to acutely avert impending seizures^2^. Hence, a better understanding of the informative biomarkers underlying the transition to seizures is needed.

Most research devoted to the study of seizure onset mechanisms and prior warning signals has traditionally focused on electroencephalogram (EEG). Continuous EEG, however, is impractical for monitoring over extended periods of time, especially when used in the ambulatory setting^3^. Wearable devices might offer a promising alternative, as these afford easy-to-use, close monitoring of autonomic nervous system (ANS) function without being invasive or restraining to PWE^4^. Alterations of ANS activity are known to occur frequently within multiple domains, such as electrodermal, thermal and cardiac subsystems, in relation to seizures, and show specific patterns across these parameters^5–7^. However, further research on these subsystems of the ANS and whether they may afford statistically meaningful identification of preictal periods in terms of sensitivity and specificity is needed.

Here, we investigate unimodal recordings from the ANS during multi-day, in hospital monitoring of PWE using wearable devices. The aim of this explorative study is to assess the utility of such ANS metrics in identifying seizures early, specifically delineating the preictal period in terms of sensitivity and specificity.

## Materials and methods

### Data recording

We recruited patients admitted to the long-term video-EEG monitoring unit at Boston Children’s Hospital between February 2015 and October 2018. We did not exclude infants, as we believe that this age group is especially in need of seizure detection and prediction from wearable devices and it was possible to fit the sensor to these patients. Patients wore a biosensor wristband (E4, Empatica Inc., Milan, Italy) on either left or right wrist or ankle for long-term recording during their admission. These sensors capture the ANS data including electrodermal activity (EDA), heart rate (HR), and temperature (TEMP). Video and EEG recordings were reviewed by board-certified epileptologists, blinded to ANS signals, to determine the seizure type, ictal EEG localization, and EEG seizure onset and offset. Written informed consent was obtained from all participants or their guardians enrolled in the study. We received approval from the Boston Children's Hospital Institutional Review Board and all research was performed in accordance with relevant guidelines/regulations. All epileptic seizures occurring in a patient were considered (Table 1).

**Table 1:**
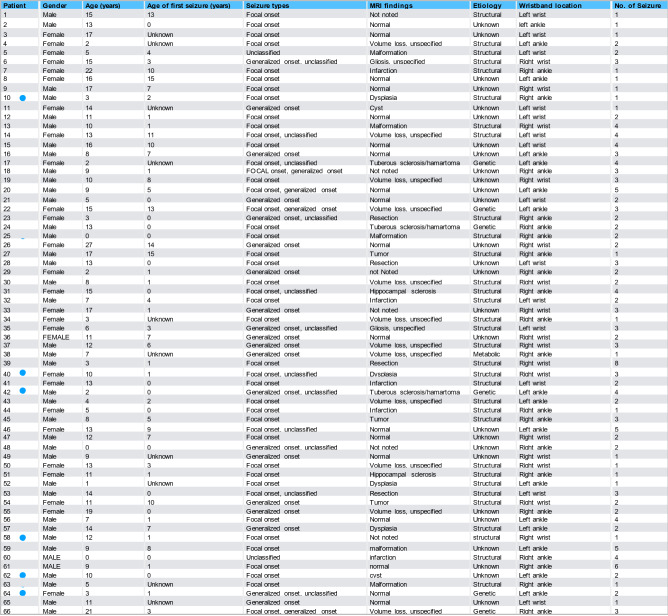
Summary of patient characteristics.

Blue dots next to patient ID indicate a significantly increased EDA signal entropy preictally (analysis for 30-s segments).

### Data analysis and statistics

**Figure 1:**
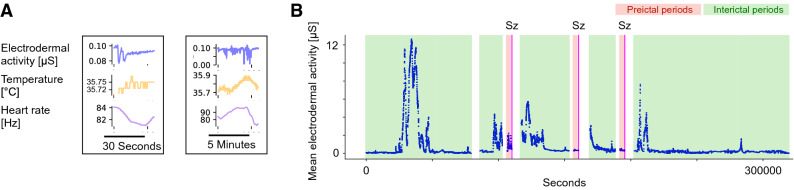
Multimodal wristband sensor data obtained during long-term epilepsy monitoring. (**A**) Example of a 30-s (left) and 5-min (right) data segments from one patient containing electrodermal activity (EDA), temperature (TEMP) and heart rate (HR). (**B**) Time course of mean EDA data from one patient. Magenta vertical lines indicate seizures (Sz), green boxes indicate periods classified as interictal, red boxes indicate periods classified as preictal.

The aim of the current study is to evaluate whether there is a systematic difference in markers recorded with a wearable device during the preictal period compared to the interictal period. For this purpose, data was analyzed in segments, for which we explored a range of either 30 s or 5 min duration (Fig. 1A). A segment was assumed preictal if it occurred between 61 min and 1 min prior to a seizure, leaving a buffer period of 1 min prior to seizure onset (Fig. 1B, red boxes). This preictal period definition was assumed to be commensurate with other research investigating the preictal period using EEG and ECoG^8–10^ and to account for potential small ambiguities in determining the exact seizure onset between the EEG and wristband. The duration choice is also based on research demonstrating that seizure generation likely takes place over minutes to hours^11^. Results were robust under different choices of the preictal period, e.g. 5-min buffer period to seizure onset and 30 min preictal period duration (see below). A segment was classified as interictal if it occurred at least 2 h prior to or after any seizure (Fig. 1B, green boxes). Similar to previous research^8,9^, we limited our analysis to lead seizures and considered seizures only if they were separated by at least 2 h. We thus excluded intervals directly after the onset of a seizure or when many seizures occurred in rapid progression in order to not bias our analyses with seizure effects and with postictal period findings^12^. To allow for stable recording conditions, we excluded data from the first and last hour.

EDA and TEMP are both recorded at 4 Hz, HR at 1 Hz. For each segment of EDA, HR, and TEMP data, the mean, variance, and entropy were calculated. Entropy is a measure that has been used to describe the degree of complexity within a time series^13^. We chose signal entropy as a potential signal marker of interest, since entropy has been shown to increase prior to certain state changes in neural systems before^14^. Related to epilepsy entropy was previously used for example in EEG^15^ and ECG^16^ data. Entropy was calculated for each segment as H = −*p* log *p*, with log denoting the logarithm to base 2 and *p* being the probability density obtained by binning the data into *n* bins. We found that results were robust for different bin numbers (n = 16, 32 bins). We thus report results for n = 32 bins.

Next, distributions of metrics (mean, variance, entropy) from the assumed preictal period were compared with the remaining data using the receiver-operating-characteristic (ROC)^17^, which allows assessing the separability in terms of sensitivity and specificity. The area under the ROC curve, Area (ROC), was calculated relative to the case of identical distributions (i.e. relative to 0.5). Thus, the value of the Area (ROC) is positive/negative when an increased/decreased indicator is indicative of a preictal period^10^.

Subsequently, seizure time surrogates were used to assess the statistical validity of any finding^11^. For each subject, a total of 100 different surrogate sets of randomized seizure onset times were created by random permutation of preictal and interictal periods. Considering a significance level of 5%, an increased/decreased indicator during the preictal period can then be considered significant, if Area (ROC) is larger/smaller than zero for the original seizure times and if Area (ROC) is larger/smaller than 95/5% of the 100 seizure time surrogates^10^. Finally, to determine whether, across patients, an indicator was increased or decreased during preictal periods, we also performed a two-sided Wilcoxon signed-rank test on the Area (ROC) values that had passed the surrogate test. Data analysis was performed using Python (version 2.7).

### Ethical approval

We confirm that we have read the Journals position on issues involved in ethical publication and affirm that this report is consistent with those guidelines.

## Results

We analyzed multi-modal signal data related to ANS function recorded from wristbands of 66 patients (9.9 ± 5.8 years; mean ± std; see Table 1 for complete patient characteristics) during long-term video-EEG monitoring. Figure 1A illustrates the data, which includes EDA, TEMP, and HR, from one patient. We analyzed data in 30-s long, non-overlapping windows; we also confirmed that results were robust under choice of a different window length (5 min). Figure 1B shows the time course from one patient where red and green boxes indicate assumed pre- and interictal periods, respectively. We report results for an assumed preictal period duration of 60 min; similar results were obtained when a preictal period of 30 min was assumed or when a 5- instead of a 1-min gap between the end of the preictal period and seizure onset was assumed. We chose both segment lengths, different preictal time periods and gaps as the relevant time scale has not been defined and to validate results.

**Figure 2:**
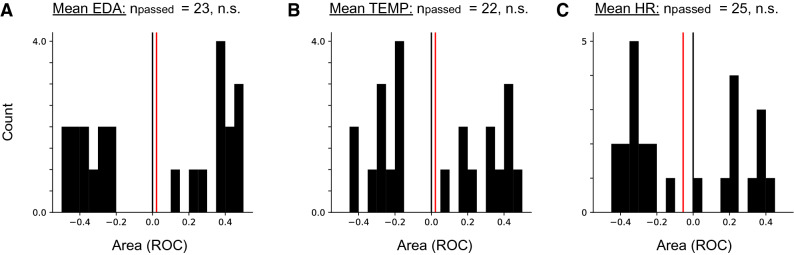
No indication of a systematic change of mean EDA, TEMP or HR during preictal periods. Distribution of values of Area (ROC) for patients that passed the surrogate test (n_passed_) for mean EDA (**A**), mean TEMP (**B**) and mean HR (**C**). Red vertical lines indicate the mean of distributions, which is not significantly different from zero in any of the cases. Results shown are for analyses on 30-s segment lengths.

As a first step, we calculated mean and variance for each sensor data stream per segment. Mean values varied widely over the recording period (Fig. 1B) and no pattern specific to the preictal period was visually detectable. Employing the receiver-operator-characteristic (ROC) yielded both increased and decreased markers that passed the surrogate seizure time test. Wilcoxon signed-rank test on the Area (ROC) values revealed no significant trend that would suggest increased or decreased mean values during preictal periods for any of the variables across patients (Fig. 2, analysis of mean values per data stream for 30-s segments). Similarly, no significant difference indicative of a coherent change across patients was observed for signal variance independent of segment length. Thus, while a subset of patients passed the surrogate test, this therefore does not preclude that these metrics change in a patient-specific way during the preictal period. Across patients there is no conclusive trend indicative of an increase or decrease in our data in terms of signal mean and variance.

**Figure 3:**
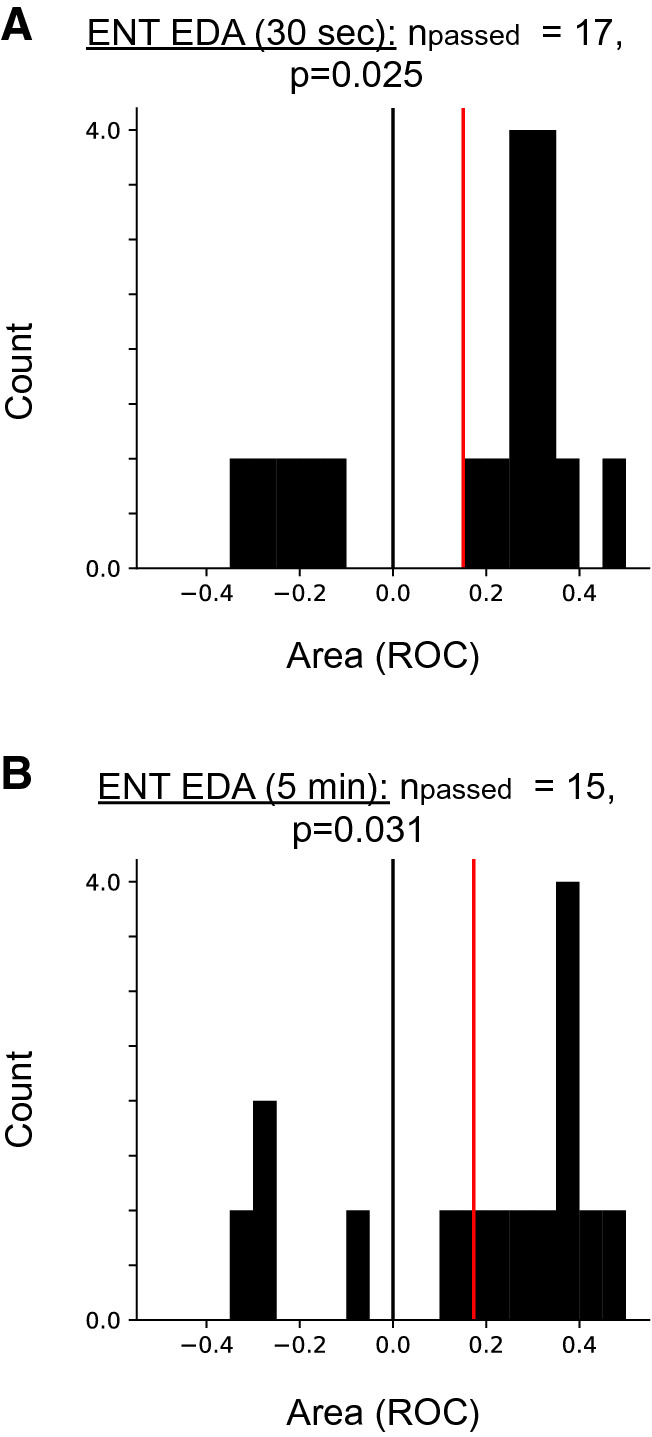
Indication of a systematic increase in EDA signal entropy in a small subset of patients during preictal periods. Distribution of values of Area (ROC) for patients that passed the surrogate test (n_passed_) for EDA signal entropy analyzed on 30-s (**A**) or 5-min segment data (**B**).

We then investigated signal entropy, a unimodal measure that has been used to quantify the complexity of a signal^13^. Further, signal entropy has also been observed to increase prior to certain state changes in neural systems^14^. In our data, entropy of HR and TEMP showed no significant trend across patients. For entropy of the EDA signal we observed significantly higher values in pre- than in interictal periods for the small subset of patients that passed the surrogate test. The increase was observed independently of whether entropy was calculated from 30-s (Fig. 3A) or 5-min segments (Fig. 3B). Additional analyses furthermore revealed that EDA entropy results were robust under different numbers of bins (16 and 32) used to calculate entropy. In summary, we observed no conclusive difference for mean and variance of the data streams. A trend to increased EDA signal entropy in the preictal period was observed in a small subset of patients. See Table 2 for a results summary.

**Table 2:** Summary of surrogate test results for all measures (analysis for 30-s segments).

Modality	Measure	Count (n_passed_)	*p* value
EDA	Mean	23	0.584
	Variance	15	0.088
	Entropy	17	0.025
HR	Mean	22	0.548
	Variance	24	0.346
	Entropy	24	0.493
TEMP	Mean	25	0.382
	Variance	26	0.501
	Entropy	23	0.484

## Discussion

The search for markers indicative of an impending seizure has a long history in epilepsy^11^. Our analysis is motivated by the benefits related to timely warning of seizures, including the ability to treat earlier or to modify activities accordingly^1,2^. Despite much effort, the current inability to predict seizures constitutes a major burden for PWE, their families, and healthcare providers^1^. Therefore, methods to assess seizure risk, to identify seizures, and to provide a warning prior to seizures, are highly desirable for patients and clinicians, in particular with non-invasive, non-stigmatizing peripheral sensors. Detection of meaningful changes with wearable wristband sensors prior to seizure onset might help avoid limitations associated with the more complex setup of EEG-based approaches^14^.

While most work in this domain has traditionally focused on EEG, ECoG and ECG, larger datasets from wearable devices might be crucial for broader application of such a method. As a relatively novel and under-explored data modality, the primary aim of this study was not to develop a fully-functioning seizure forecasting system, but to statistically assess the possibility of meaningful data features to identify seizures early, ideally prior to EEG onset. Identification of such markers may guide further investigation to establish an early-warning system and may potentially provide novel insights into the physiology of seizure generation in a different manner than traditional EEG- and ECoG-based methods. To approach this goal, we decided to use the surrogate marker approach to exploratively test which measures for each signal show relevant differences between preictal and interictal segments. We used mean and variance to capture main characteristics of the signals in the time domain. Furthermore, we calculated entropy, a measure from the information domain to infer the state of the respective ANS subsystem; entropy has previously been suggested to differentiate between ictal and non-ictal segments of intracranial EEG data^15^.

Our main finding is that simple mean or variance signal values may be insufficient to reliably distinguish pre- and interictal periods with sufficient sensitivity and specificity. Our work highlights the importance to carefully assess any data feature over long periods of time in order to truly determine its value in terms of sensitivity and specificity. Signal entropy has previously been studied with respect to other stressors, i.e. pain or social stress that included EDA entropy in the group of predictive features that differentiate between states^18,19^. Our results cautiously suggest that EDA entropy may potentially be promising in this regard also in epilepsy, as it provides statistically meaningful results for a small subset of patients in terms of a temporal relationship between these markers and timing of seizure onset. However, further validation and investigation of the potential causes underlying these changes, e.g. if the observed changes truly reflect a physiological phenomenon or are related to some systematic data confounder or other stressor, are needed and merit further investigation. Nevertheless, if confirmed in larger patient cohorts, such markers may potentially help to devise personalized seizure risk assessment algorithms.

Changes in central nervous system physiology may precede epileptic seizures prior to their seemingly abrupt onset^20^. Slow changes may also be detected through peripheral sensors monitoring ANS function. Entropy may potentially capture some of these changes. Our analyses utilized a unique dataset comprised of long-term ANS monitoring data from a large number of patients admitted for video-EEG monitoring at the same hospital, in which the same procedures were followed. Thus, this study complements a multicenter study that evaluated ANS data on seizure detection^21^. Pediatric patients are combined with adult patients and several devices are used, but the focus is on convulsive seizures. Interestingly, the results of both studies show the importance of EDA. We conclude that in the context of seizure prediction and detection, a closer analysis of EDA signals can spur novel research ideas, even with less frequently used analytical approaches^22^.

The effects of seizures on different subsystems of the ANS have been shown for various seizure types^5,23,24^. The combination of subsystems of the ANS and evaluation of markers to describe seizure-related changes within and across subsystems permits deeper insights into complex ANS activity patterns^25^. Our results may support the hypothesis that seizures are related to ANS changes and that the pre-seizure state, as a central stimulus, alters the central control of ANS activity. This may potentially underlie the changes in the EDA signal observed in some patients. It is also likely that seizures arising from some brain regions (e.g. the temporal lobe) engage the ANS more than seizures arising from other brain regions^26^. In our dataset, a lesion such as one in mesial temporal sclerosis, was rare as it is not common in pediatric patient populations (Table 1; only two documented hippocampal sclerosis lesions). Thus, while in principle it is possible that changes in the ANS are more frequent with seizures originating or spreading in certain areas of the brain, (e.g. seizures of temporal lobe origin), our data did not allow a comprehensive investigation of this hypothesis due to the low number of patients and the nature of the pediatric cohort.

Another important aspect when analyzing ANS signals is that different subsystems may act on different time scales. Also, each subsystem has various regulatory processes that challenge the definition of relevant time periods to consider. Here, we chose to analyze 60 min of preictal data and verified that the results remain similar when analyzing 30 min only. Also, we varied the segment length from which the mean, variance, and entropy were calculated to validate our results. By defining these parameters, as well as parameters set during data processing, e.g. bin number, it became challenging that these standards are not set in the field. Guidelines for data collection, processing and analysis are still missing for data from wearable devices.

Results need to be interpreted in the setting of data acquisition. First, the results are based on a sample of mostly treatment-refractory PWE, which may imply that the changes in ANS functioning relate to the refractoriness of medication. Therefore, generalizability to well-controlled epilepsy patients may require further work, such as validation on additional data sets. Additionally, our dataset was recorded in a hospital setting, and seizure forecasting in everyday life will face alternate challenges. At the moment, this setup allows for a controlled situation. Furthermore, the hospital setting causes additional stressors, e.g. sleep deprivation, changes in medication, and increased seizure likelihood. These factors cannot be completely controlled within the current dataset and will need to be taken into account when considering real-life applications.

We only performed minimal preprocessing of the data to remain close to real-life recording conditions. Moreover, anti-seizure drugs may have been weaned during monitoring, and therefore we cannot rule out confounding effects of anti-seizure drugs adjustments^27^. To describe ANS activity we selected three continuous measures, EDA, HR and TEMP. This selection excluded other potentially relevant measures, such as respiratory rate. The reason for the selection was based on the Empatica E4 sensor used for data recording, which collects the studied modalities. We favored one device allowing for data synchronization over additional measures. The sensor allows for long-term data recordings but with comparably low sampling rates. Another challenge is managing artifacts, such as sensor location or movement artifacts. We considered them to incur similarly in inter- and pre-ictal data and therefore did not exclude artifact-affected segments. Furthermore, one has to carefully consider other causes for the observed changes in EDA signal entropy. For example, the possibility that changes in signal quality due to a loosely fitting wristband contributed to the observed effects, cannot be completely ruled out. Future confirmation of the observed findings in larger patient cohorts are thus essential. Of note, we did not compare seizure type specificity with ANS changes. Instead, we included all seizure types and combined different seizure types from one patient into our analysis. We were specifically interested in broad markers indicative of an impending transition to seizures and favored the large dataset to detect an entry point to further develop biomarkers with the potential to contribute to seizure forecasting. Lastly, as also inherent in similar, we cannot rule out selection and information bias. But through outlining enrollment, patient selection processes, and data acquisition, we tried to generate reproducible and transparent results while acknowledging these shortcomings.

In summary, we used a unique dataset to assess potential ANS markers that would be informative of an approaching epileptic seizure in terms of significant sensitivity and specificity. When applying statistical testing using surrogate seizure times, mean and variance values of EDA, TEMP and HR did not exhibit a consistent trend across patients. While findings demand further validation and research on the underlying causes, changes in EDA signal entropy may be observed in a small subset of patients and potentially afford searching for more personalized seizure risk markers. Clinically, robust state changes detectable from wearable wristband sensors may provide new opportunities for seizure risk assessment and forecasting based on non-invasive, easy-to-use devices.
